# Does HIF-PHI increased risk of gastrointestinal hemorrhage in patients with renal anemia: a review of cases reported to the U.S. Food and drug administration adverse event reporting system

**DOI:** 10.1080/0886022X.2021.1952881

**Published:** 2021-07-27

**Authors:** Yangzhong Zhou, Qidong Ren, Rongrong Hu, Ke Zheng, Yan Qin, Xuemei Li

**Affiliations:** Department of Nephrology, Peking Union Medical College Hospital, Beijing, China; School of Medicine, Tsinghua University, Beijing, China

Dear Editor,

The efficacy of hypoxia-inducible factor prolyl hydroxylase inhibitors (HIF-PHIs) in correcting renal anemia, including in special populations, has been demonstrated in several randomized controlled trials (RCTs). However, data regarding the safety profiles in these agents are lacking, especially in the real-world setting.

We analyzed safety signals of HIF-PHIs in the US Food and Drug Administration (FDA) adverse event Reporting System (FAERS), a useful approach to monitor rare adverse events *via* assessment of disproportionality. We referred to OpenVigil 2.1 for data queries and extraction [1], including Daprodustat and Roxadustat between January 2018 and September 30, 2020. A total of 2,656,846 reports were submitted to FAERS, and 30 reports on HIF-PHIs were recorded (Table 1). Among them, 25 were from Daprodustat and 5 from Roxadustat. The median age was 64, which was numerically similar to those in clinical trials [2–4]. HIF-PHIs were the primary or secondary suspect in most reports (93.3%), with the indication of nephrogenic anemia. Death of life-threatening cases constituted 5 (16.67%) of them, and 28 (93.33%) of them required hospitalization.

**Table 1.** Clinical characteristics of adverse events related to HIF-PHIs.

	AEs in HIF-PHIs
Drug	
Daprodustat	25
Roxadustat	5
Gender	
Male	16
Female	12
Missing	2
Age	
<65	13
≥65	13
Missing	4
Year	
2018	15
2019	11
2020	4
Outcomes	
Death	2
Life-threatening	3
Hospitalization	23
Other serious	5
Role code	
Primary suspect	4
Secondary suspect	24
Concomitant	2
System organ class (SOC)	
Vascular disorders	8
Infections and infestations	7
Gastrointestinal disorders	3
Injury, poisoning, and procedural complications	3
Metabolism and nutrition disorders	2
Blood and lymphatic system disorders	1
Eye disorders	1
General disorders and administration site conditions	1
Immune system disorders	1
Neoplasms benign, malignant, and unspecified (incl cysts and polyps)	1
Nervous system disorders	1
Renal and urinary disorders	1

Eight reports were related to vascular disorders. Of note, gastrointestinal (GI) hemorrhage was filed in four of them, together with two cases of hemorrhoidal hemorrhage, one case of hemorrhagic stroke, and one case of epistaxis. All four of these cases with GI hemorrhage were female and hospitalized. Disproportionality analysis showed that GI hemorrhage was significantly more common in reports on HIF-PHIs compared to those filed for other drugs, with a reporting odds ratio (ROR) of 29.27 (95% CI 10.21–83.88; *p* < 0.05).

In the FGCL-4592-808 trial, gastrointestinal hemorrhage was reported in the Roxadustat group (1/101) [3]. While in the other trials or literature of HIF-PHIs, reports on GI hemorrhage were not found [2,4]. We performed a similar analysis for EPO, by searching epoetin alfa, epoetin beta, darbepoetin alfa, and methoxy polyethylene glycol-epoetin beta in FAERS. We found 34 cases of GI hemorrhage in total, with a ROR of 0.77 (95% CI 0.55–1.08; *p* > 0.05). Compared to EPO, the ROR of GI hemorrhage in HIF-PHIs was 37.81 (95% CI 12.52–114.20; *p* < 0.05).

However, our analysis based on FAERS data has several limitations to be noted. Firstly, the ROR significance didn’t imply a causal relationship between the drug and adverse events. Further cohort studies or long-term follow-ups of the trial patients are in need to support clinical practice. Secondly, data from the records in FAERS were not complete enough to inform potential risk factors. Based on the information about concomitant medications, most patients with GI hemorrhage in our analysis also used aspirin, clopidogrel, or heparin, etc. But it’s difficult to understand their role in GI bleeding, due to the lack of clinical details (dose, time, type of bleeding, etc.). Thirdly, the sample size in this analysis was small. After the approval of HIF-PHIs in several countries, more reports could be expected to increase the power of this kind of analysis.

To summarize, our descriptive exploration based on FAERS data might suggest an increased risk of GI hemorrhage when using HIF-PHIs to treat renal anemia. It’s not clear whether this is a class effect or not. The potential mechanism between HIF and GI bleeding was also unknown. Previous studies suggested that abnormal angiogenesis in the GI tract could be regulated by HIF, by directly targeting the VEGF pathway [5]. Future clinical and biological studies are in need to provide more evidence.
